# Long-term symptoms after SARS-CoV-2 infection in a cohort of people living with HIV

**DOI:** 10.1007/s15010-024-02288-9

**Published:** 2024-05-03

**Authors:** Melania Degli Antoni, Giovanni Maifredi, Samuele Storti, Giorgio Tiecco, Marco Di Gregorio, Benedetta Rossi, Cinzia Gasparotti, Emanuele Focà, Francesco Castelli, Eugenia Quiros-Roldan

**Affiliations:** 1 Unit of Infectious and Tropical Diseases, Department of Clinical and Experimental Sciences, ASST Spedali Civili Di Brescia and University of Brescia, Brescia, Italy; 2 ATS Brescia (Brescia Health Protection Agency), Brescia, Italy

**Keywords:** HIV, PLWH, COVID-19, Long-COVID, Post-COVID-19 conditions, Post-acute sequelae of SARS-CoV-2

## Abstract

**Background:**

Our Hospital in Northern Italy assists 3817 people living with HIV (PLWH) and has faced the impact of COVID-19. Little is known about the impact of HIV infection on the risk of post-COVID-19 conditions (PCCs) onset. We aim to assess the incidence of PCC in PLWH and the factors associated with its occurrence.

**Methods:**

We performed a retrospective, observational study including all PLWH > 18 years registered in the Brescia Health Protection Agency database, assessing SARS-CoV-2 burden, vaccination status, socio-demographic, and viro-immunological parameters from February 2020 until May 2022. Persistence of self-reported symptoms (clustered into gastrointestinal, respiratory, osteo-muscular, and neuro-behavioral symptoms) was evaluated after 3 months by a telephone-administered questionnaire. We estimated the associations between all variables and outcomes through univariate and multivariable logistic models.

**Results:**

In the study period, 653 PLWH were diagnosed with SARS-CoV-2 infection (17.1%). We observed 19 (2.9%) reinfections, 71 (10.9%) hospitalizations, and 3 (0.5%) deaths. We interviewed 510/653 PLWH (78%), and 178 (PCCs prevalence 34.9%; CI 95% 30.7–39.2) reported persistent symptoms. Asthenia/fatigue was the most reported symptom (60/178), followed by muscular pain (54/178). In the multivariate regression model, there was a lower risk of PCCs in males respect to females (adjusted OR = 0.64; CI 95% 0.99–3.66), while hospitalization during acute infection was associated with an increased the risk of PCCs (adjusted OR = 1.9; CI 95% 0.99–3.66). Notably, no viro-immunological variable modified the PCCs risk onset.

**Conclusions:**

Our study highlights a substantial prevalence of PCCs among PLWH, three months post-SARS-CoV-2 infection, independent of viro-immunological features or vaccination status.

**Supplementary Information:**

The online version contains supplementary material available at 10.1007/s15010-024-02288-9.

## Introduction

Severe acute respiratory syndrome coronavirus 2 (SARS-CoV-2) was responsible for the coronavirus disease of the 2019 (COVID-19) pandemic and for a new clinical entity called “long-COVID”, “post-acute sequelae of SARS-CoV-2” (PASCs) or “post-COVID-19 conditions” (PCCs) [1]. The World Health Organization (WHO) defined the PCCs as a condition which occurs usually 3 months after the onset of COVID-19 in individuals with a history of probable or confirmed SARS-CoV-2 infection, and with symptoms lasting for at least 2 months that cannot be explained alternatively [2]. This new syndrome affects over 10 millions of people worldwide engendering another global health threat [3].

The mechanisms behind the pathogenesis of PCCs remain unclear. Several pathophysiological models have been proposed including SARS-CoV-2 persistence in “reservoirs”, cell dysmetabolism, autoimmunity caused by molecular mimicry, host-microbiome changes, and SARS-CoV-2 reactivations [4]. The symptoms of PCCs are likely caused by organ damage induced by the acute infection phase, mostly affecting the respiratory, neurological, cardiovascular, and musculoskeletal systems, although distinct long-lasting inflammatory pathways have also been hypothesized [5]. Certainly, differences in the virus variant, SARS-CoV-2 vaccination status, and host response likely contribute to the risk, severity, and duration of PCCs [5].

Addressing risk factors and clinical manifestations of PCCs in population groups will help identify patients who may require close monitoring after recovery from acute COVID-19 [6]. Immunodeficiency has been recognized a crucial risk factor for severe COVID-19 since the early COVID-19 pandemic [7].

Considering the high burden of comorbidities linked to the immune-activation, especially those not receiving an appropriate antiretroviral therapy (ART), people living with HIV (PLWH) are considered to be more vulnerable to severe SARS-CoV-2 infection, and, potentially, to PCCs [8].

Therefore, this study aims to investigate the prevalence, clinical symptoms, and risk factors for PCCs among PLWH of Northern Italy province highly affected by COVID-19.

## Material and methods

### Study design and participants

This is an observational retrospective cohort study that included PLWH aged 18 years or older followed at the Unit of Infectious Diseases of the Spedali Civili General Hospital of Brescia, Italy. These individuals had tested positive for SARS-CoV-2 via real-time polymerase chain reaction (RT-PCR) on nasopharyngeal swabs from February 15th, 2020, to May 31st, 2022. Written informed consent was obtained from every participant. No exclusion criteria were considered.

## Data collection

We conducted data matching between the electronic health records of our Unit and the Brescia Health Protection Agency database, formerly known as the Brescia Local Health Agency database (BLHADB). We collected demographic, epidemiological, clinical, and laboratory data. Vaccination coverage data were also recorded. To assess COVID-19 prevalence, we considered infected with SARS-CoV-2 all people with a confirmed nasopharyngeal swab positive for SARS-CoV-2 by RT-PCR with a European Emergency Use Authorization (EUA). To assess the potential impact of SARS-CoV-2 variants, we divided the study period into two phases: pre-Omicron (from February 15th, 2020, to December 31st, 2021) and Omicron period (from January 1st, 2022, to May 31st, 2022). SARS-CoV-2 infections with the Omicron variant were defined based on the period of predominance, as reported by our National Surveillance Institute (Istituto Superioriore di Sanità, Epicentro ISS) that provides periodic reports monitoring the circulating variants prevalence in Italy [9]. In particular, as highlighted by the 16th report, by January 10th 2022 almost 90% of the isolated variant in Lombardy region, where this study was conducted, were Omicron [10]. We considered fully vaccinated patients who had received 3 doses of the vaccines routinely used in Italy (BNT162b2, mRNA-1273, and ChAdOx1). Of note, ChAdOx1 was exclusively used for the first and the second doses. In case of Ad26.COV2.S vaccine administration as first dose, the second one was considered as a booster dose. Thus, individuals in this group were considered fully vaccinated following the administration of the second dose.

## PCCs definition

The diagnosis of PCCs was defined based on the WHO criteria [2]. Symptoms related to PCCs were those of new onset, or previously reported but with a significant worsening, that could not be attributed to alternative causes [2]. The following symptoms were considered: breathlessness (dyspnea), weakness and tiredness (asthenia/fatigue), joint pain (arthralgia), muscle pain (myalgia), head pain (headache), sore throat, abdominal pain, diarrhea, bumps or redness on the skin (rash), numbness, eye-related symptoms (eye pain, itching, foreign body sensation, redness, watery eyes, eye discharge), memory loss or impairment, reduced ability to think and concentrate (poor concentration).

We evaluated the occurrence of PCCs symptoms based on their timing relative to the acute infection: less than 4 weeks, between 4 and 12 weeks, or more than 12 weeks from the acute infection.

Resident doctors in Infectious and Tropical Diseases working at the Spedali Civili General Hospital of Brescia, Italy conducted telephone interviews using prespecified questionnaires (see Supplementary Appendix) to systematically collect a comprehensive list of symptoms.

## Ethics

This study was performed in accordance with the Helsinki Declaration of 1975 as revised in 2013, and it was approved by the local ethical committee of the Spedali Civili General Hospital of Brescia (approval code NP 5477, local ethical committee approval date 10th August 2022).

### Statistical analysis

The sociodemographic and clinical characteristics of study participants were described using numbers and proportions of the overall sample and specific subgroups (hospitalized/non-hospitalized PLWH, PLWH with long-COVID/without long-COVID). The non-parametric Mann–Whitney test and the chi-square test were used to investigate the statistical significance of differences between numerical and categorical variables, respectively. Possible risk factors for persistent COVID-19 symptoms over 90 days were identified. The odds ratios (ORs) for persistent COVID-19 symptoms were calculated using both univariate and multivariate logistic regression. The included covariates were SARS-CoV-2 vaccination, infection period, years of HIV infection, HIV-RNA at the time of SARS-CoV-2 infection, nadir CD4^+^ T cells (stratified by terciles), CD4^+^, CD8^+^ T cells and CD4^+^/CD8^+^ ratio at the time of SARS-CoV-2 infection (stratified by terciles), sex, age, number of comorbidities and ethnicity. Sensitivity analyses were performed considering age and years of HIV infection as both continuous and grouped variables (< 65, 65–79, 80 for age; in continuous groups of 5 or 10 years for years of HIV infection). In accordance to ethical practices for the dissemination of scientific data, in tables where there was a value of 0, 1 or 2, we reported less than three (< 3). We computed the cumulative percentage of SARS-CoV-2 infections during the study period stratified by presence and absence of long-COVID to illustrate the evolution of SARS-CoV-2 infection in PLWH over time. The confidence intervals were computed at the 95% level. All statistical tests.

were two-sided, assumed a level of significance of 0.05 and were performed using Stata 17 software (StataCorp. 2021. Stata Statistical Software: Release 17. College Station, TX, USA: StataCorp LLC).

## Result

### Study population, vaccination coverage and SARS-CoV-2 prevalence

**Fig. 1:**
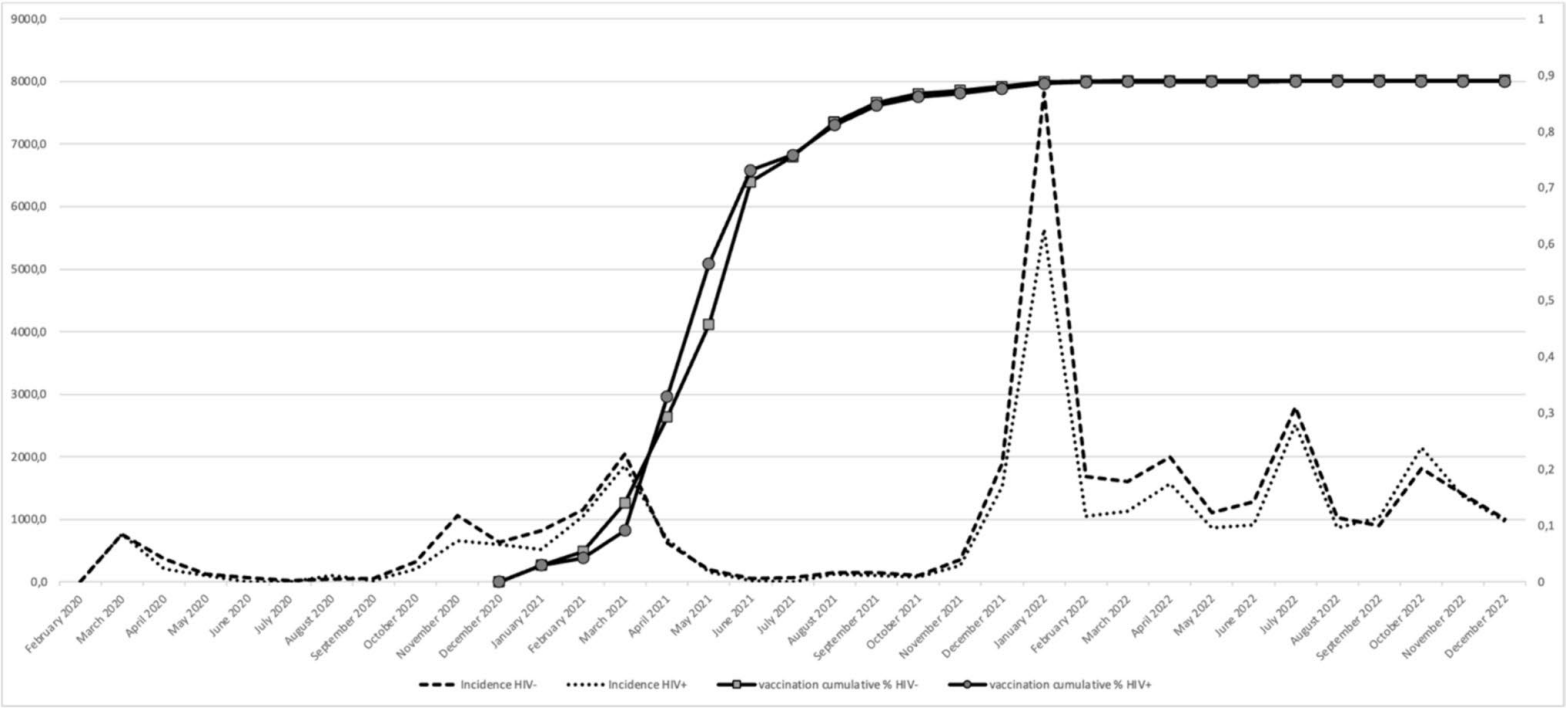
Incidence of SARS-CoV-2 infection and SARS-CoV-2 vaccination (at least one dose) in PLWH and non-PLWH

A total of 1,004,210 individuals over 18 years old belong to the Brescia Health Protection Agency (HPA) area. Among them, 3817 (0.38%) are PLWH. The monthly trend of SARS-CoV-2 incidence and vaccination coverage per 100,000 inhabitants (at least one dose) between February 2020 and December 2022 comparing PLWH to the general population is shown in Fig. 1. The incidence curves for SARS-CoV-2 infection were observed to closely overlap between PLWH and those without HIV, both experiencing a peak in infections concurrent with the emergence of the Omicron variant. Moreover, the trend in COVID-19 coverage was similar between PLWH and individuals without HIV infection.

Table 1 summarize the characteristics of PLWH residing in the Brescia HPA area. The median age was 54 years (IQR 46.5–58.9), with a predominant prevalence of individuals aged 50–69 years (56.1%). The majority of PLWH were male (71.2%) and Italians (86%). SARS-CoV-2 infection was diagnosed in 17.1% (653/3817) of PLWH population, with only 19 cases (2.9%) of reinfection. Hospitalization for COVID-19 was required for 71 (10.9%) patients, while 3 (0.5%) deaths were observed.

**Table 1:** Characteristics of PLWH population attending Brescia HPA and the impact of SARS-CoV-2 infection

PLWH, n	3817
Age, median (IQR)	54 (46.5 – 58.9)
Age, category	
18–49, n. (%)	1311 (34.3)
50–69, n. (%)	2140 (56.1)
> 70, n. (%)	366 (9.6)
Male, n. (%)	2717 (71.2)
Comorbidities	
None (%)	1679 (44)
1 (%)	933 (24.4)
2–3 (%) > 3 (%)	932 (24.4) 273 (7.2)
Citizenship	
Italians, n. (%)	3284 (86)
Non-Italians, n. (%)	533 (14)
SARS-CoV-2 infection, n. (%)	**653 (17.1)**
Pre-omicron period, n. (%)	252 (38.6)
Omicron period, n. (%)	401 (61.4)
SARS-CoV-2 re-infection, n. (%)	19 (2.9)
COVID-19 hospitalization, n. (%)	71 (10.9)
COVID-19 deaths, n. (%)	3 (0.5)

The 39% of patients (252) got infected between the beginning of the pandemic and November 2021, while 401 (61.4%) PLWH got infected during the Omicron period. In contrast, during the pre-Omicron period, 49 patients were hospitalized (69% of admissions), while during the Omicron period, 22 patients were admitted (31% of admissions), showing a statistically significant difference between the two groups (p < 0.001).

## Post-COVID conditions symptoms in PLWH

A total of 510 PLWH (78.1%) consented to the telephone interview to identify PCCs symptoms after SARS-CoV-2 infection. Among these, 178 patients (34.9%; CI 95% 30.7–38.2) exhibited at least one of the symptoms defining PCCs, while 332 (65.1%) did not develop symptoms following the resolution of SARS-CoV-2 infection.

The sociodemographic, clinical, and viro-immunological characteristics of the PLWH infected with SARS-CoV-2, both with and without PCCs, who answered the questionnaire, are presented in Tables 2 and 3. Similar distributions were observed between the two groups across all considered variables, except for hospitalization during acute SARS-CoV-2 infection, that was more frequent in patients with PCCs (14% vs 8%; p = 0.048). Additionally, there were no differences between the two groups in terms of viro-immunological profiles at the time of infection or serological status for HBV, HCV, CMV, and Toxoplasma.

**Table 2:** Sociodemographic and clinical characteristics of PLWH with SARS-CoV-2 infection

	Total, N. (%) 510	PLWH with PCCs, N. (%) 178	PLWH without PCCs, N. (%) 332	*p value*
Sex				
Male	359 (70.39)	121 (67.98)	238 (71.69)	0.382
Female	151 (29.61)	57 (32.02)	94 (28.31)	
Age (median, SD)				
Ethnicity	52.55 (10.81)	53.64 (9.78)	52.05 (11.26)	0.093
Italians	461 (90.39)	166 (93.26)	295 (88.86)	0.108
Foreigners	49 (9.61)	12 (6.74)	37 (11.14)	
Sexual habits				
Heterosexual	268 (52.55)	91 (51.12)	177 (53.31)	0.747
Homosexual	123 (24.12)	47 (26.41)	76 (22.89)	
Other	25 (4.9)	7 (3.93)	18 (5.42)	
Unavailable	94 (18.43)	33 (18.54)	61 (18.38)	
Antiretroviral therapy ongoing				
2NRTI + INI	183 (35.88)	67 (37.64)	116 (34.94)	0.768
2NRTI + NNRTI	51 (10)	15 (8.43)	36 (10.84)	
2NRTI + PI	21 (4.12)	6 (3.37)	15 (4.52)	
NRTI + INI	175 (34.31)	64 (35.96)	111 (33.43)	
NNRTI + INI	38 (7.45)	16 (8.99)	22 (6.63)	
INI + PI	22 (4.31)	5 (2.81)	17 (5.12)	
Others	20 (3.92)	5 (2.81)	15 (4.52)	
Comorbidities				
None	334 (65.49)	113 (63.48)	221 (66.57)	0.367
1	123 (24.12)	49 (27.53)	74 (22.29)	
> 2	53 (10.39)	16 (8.99)	37 (11.14)	
Comorbidities				
Hypertension	82	29	53	0.923
Diabetes mellitus	30	10	20	0.853
Obesity (BMI > 30)	20	9	11	0.334
Cardiovascular diseases	47	14	33	0.440
Active onco-hematologic disease	10	5	5	0.312
Onco-hematologic disease within 5y	33	14	19	0.349
Immunodeficiency	< 3	< 3	< 3	0.299
Chronic renal failure	18	5	13	0.519
Bronchopneumopathy	12	4	8	0.918
Unavailable	4	< 3	4	0.142
Vaccination for SARS-CoV2				
Unvaccinated	236 (46.28)	89 (50)	147 (42.28)	0.215
Incomplete vaccination	129 (25.29)	37 (20.79)	92 (27.71)	
Fully vaccinated	145 (28.43)	52 (29.21)	93 (28.01)	
Hospitalized for COVID-19	53 (10.39)	25 (14.05)	28 (8.43)	**0.048**
Period of infection				
Pre-Omicron	201 (39.41)	76 (42.69)	125 (37.65)	0.266
Omicron	309 (60.59)	102 (57.31)	207 (62.35)	

*PLWH* People living with HIV, *PCCs* Post-COVID19 conditions, *NRTI* nucleoside reverse transcriptase inhibitors, *INI* integrase inhibitors, *NNRTI* non-nucleoside reverse transcriptase inhibitors, *PI* protease inhibitors

*p* value in bold underlines the statistically significant association between variables

**Table 3:** Viro-immunological characteristics of PLWH with SARS-CoV-2 infection

	Total, N. (%) 510	PLWH with PCCs, N. (%) 178	PLWH without PCCs, N. (%) 332	p value
HCV-Ab				
Available	503	177	326	
Positive	144 (28.63)	52 (29.38)	92 (28.22)	0.496
Negative	359 (71.37)	125 (70.62)	234 (71.78)	
HBcAb				
Available	494	171	323	
Positive	212 (42.92)	75 (43.86)	137 (42.42)	0.758
Negative	282 (57.08)	96 (56.14)	186 (57.58)	
HbsAg				
Available	506	176	330	
Positive	30 (5.93)	8 (4.55)	22 (6.67)	0.336
Negative	476 (94.07)	168 (95.45)	308 (93.33)	
CMV Serology (IgG)				
Available	135	46	89	
Positive	122 (90.37)	43 (93.48)	79 (88.76)	0.379
Negative	13 (9.63)	3 (6.52)	10 (11.24)	
Toxoplasma Serology (IgG)				
Available	400	138	262	0.486
Positive	202 (50.5)	73 (52.89)	129 (49.24)	
Negative	198 (49.5)	65 (47.11)	133 (50.76)	
HIV-RNA at SARS-CoV-2 infection,				
< 20 cp/mL	473 (92.75)	168 (94.38)	305 (91.87)	0.299
≥ 20 cp/mL	37 (7.15)	10 (5.62)	27 (8.13)	
T-lymphocyte profile at SARS-CoV-2 infection, mean (SD)				
CD4^+^ T cells	775.11 (347.59)	753.36 (309.78)	787.99 (367.75)	0.587
CD8^+^ T cells	895.05 (583.05)	873.27 (404.74)	911.99 (698.89)	0.546
CD4^+^/CD8^+^	1.09 (1.34)	1.18 (2.34)	1.06 (0.62)	0.113
Nadir CD4^+^ T cells, mean (SD)	259.09 (191.33)	254.53 (184.84)	252.41 (185.66)	0.433

*PLWH* people living with HIV, *PCCs* post-COVID19 conditions

*p* value in bold underlines the statistically significant association between variables

**Fig. 2:**
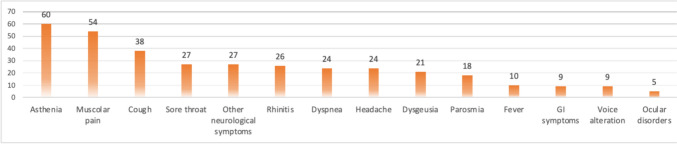
Number of PLWH who reported one or more symptoms at 3 months after SARS-CoV-2 infection

The most frequent PCCs symptoms reported during the telephone interview are shown in Fig. 2. Asthenia/Fatigue was the most reported symptom, followed by musculoskeletal pains, respiratory symptoms, and neurological symptoms.

No difference in the PCCs prevalence was observed between PLWH SARS-CoV-2 infected during the pre-Omicron period (until December 2021) and the Omicron period (from January 2022) (Table 2).

**Fig. 3:**
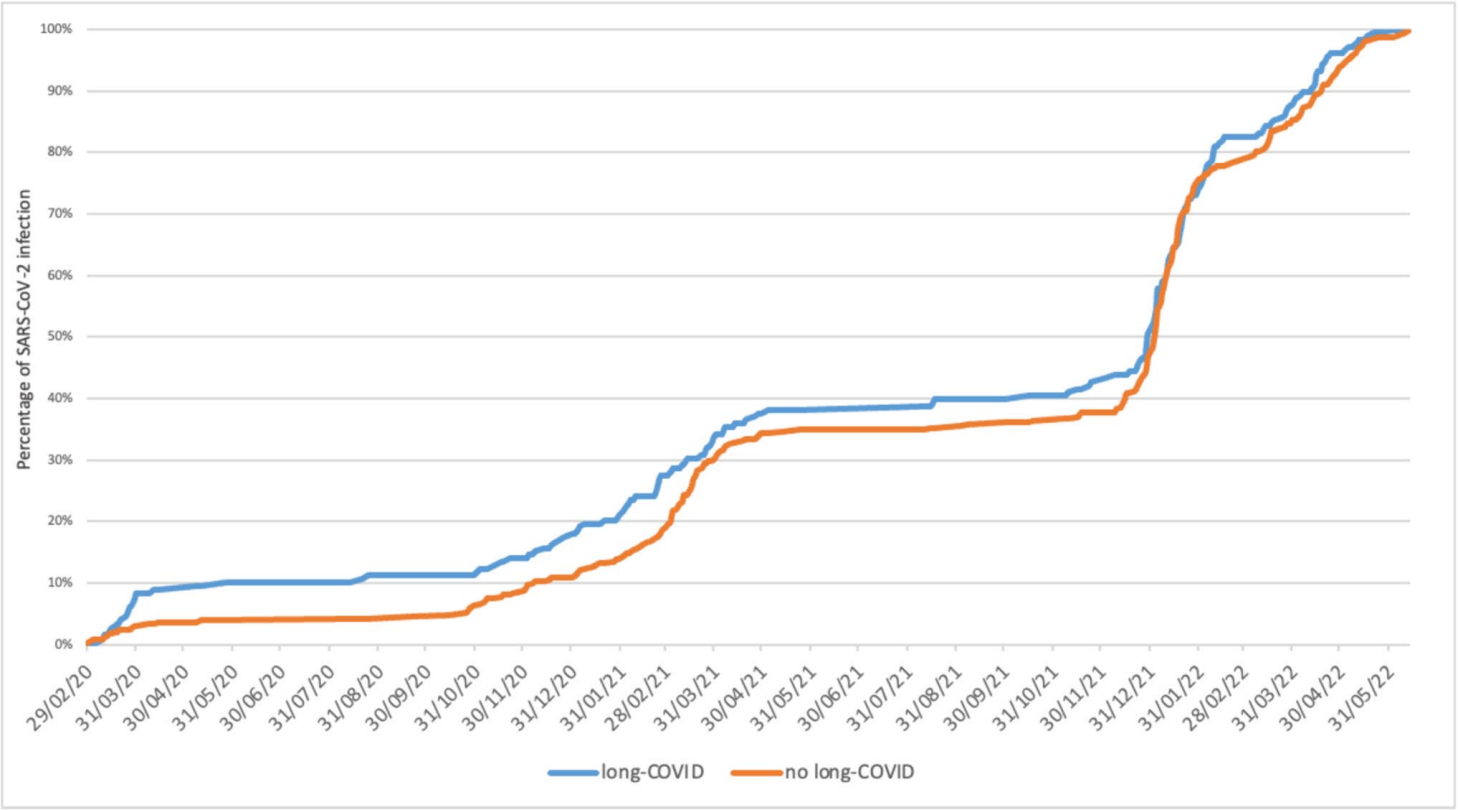
Cumulative percentage of SARS-CoV-2 infections during the study period stratified by presence or absence of long-COVID

However, analyzing the cumulative trend over time of SARS-CoV-2 infection stratified between those who developed long-COVID and those who did not, a predominant trend in the onset of long-COVID was observed among patients infected during the pre-Omicron period. However, these two curves tended to overlap during the Omicron period, as illustrated in Fig. 3.

A regression analysis was conducted to assess potential risk factors for the onset of PCCs in PLWH patients. Table 4 presents the association between demographic, clinical, and viro-immunological variables in patients affected by PCCs and those not affected. PLWH hospitalized during acute SARS-CoV-2 infection (OR: 1.77 IC 95% 1–3.1) and patients aged 55–59 years (OR: 2.11 IC 95% 1.25–3.55) were more likely to experience PCCs. However, in our adjusted model, there was a lower risk of PCCs in males respect to females (OR: 0.62 IC 95% 0.39–0.99) (Table 5).

**Table 4:** Univariate to assess potential risk factors for PCCs onset in PLWH

Variable		Odds ratio	p value	95%	CI
Sex	Female (ref)	–	–	–	–
	Male	0.838	0.382	0.565	1.245
Age, category	18–45 (ref)	–	–	–	–
	46–54	1.263	0.392	0.740	2.154
	**55–59**	**2.118**	**0.005**	**1.259**	**3.559**
	> 60	1.263	0.392	0.266	0.575
Ethnicity	Italians (ref)	–	–	–	–
	Foregneirs	0.576	0.111	0.292	1.136
Sexual habits	Hterosexual (ref)	–	–	–	–
	Homosexual	1.203	0.414	0.772	1.873
	Other	0.756	0.545	0.305	1.877
ART	2NRTI + INI (ref)	–	–	–	–
	2NRTI + NNRTI	0.721	0.342	0.368	1.414
	2NRTI + PI	0.693	0.469	0.256	1.870
	NRTI + INI	0.998	0.994	0.649	1.535
	NNRTI + INI	1.259	0.523	0.618	2.563
	INI + PI	0.509	0.204	0.179	1.443
	Others	0.693	0.666	0.131	3.668
Years of HIV infection, category	0–9 (ref)	–	–	–	–
	10–19	0.695	0.169	0.414	1.168
	20–29	1.005	0.986	0.576	1.753
	> 30	0.968	0.910	0.555	1.691
Comorbidities	No comorbidities (ref)	–	–	–	–
	Comorbidities 1–2	1.264	0.323	0.794	2.013
	Comorbidities > 2	0.827	0.591	0.414	1.654
SARS-CoV-2 Vaccination	Unvaccinated (ref)	–	–	–	–
	Incomplete vaccination	0.664	0.084	0.418	1.056
	Complete vaccination	0.924	0.717	0.601	1.419
SARS-CoV-2 Infection Period	First period (ref)	–	–	–	–
	Second period	0.810	0.267	0.559	1.174
Hospitalization for COVID-19	No hospitalization (ref)	**–**	**–**	**–**	**–**
	Hospitalization	**1.774**	**0.050**	**1.000**	**3.147**
Nadir CD4^+^ T cells	< 33 percentile (ref)	–	–	–	–
	33–66 percentile	1.100	0.677	0.702	1.723
	> 66 percentile	0.819	0.393	0.397	1.295
HIV-RNA (concurrently with SARS-CoV-2 infection)	HIV-RNA < 20 cp/mL (ref)	–	–	–	–
	HIV-RNA 20–200 cp/mL	0.859	0.717	0.381	1.943
	HIV-RNA > 200 cp/mL	0.227	0.164	0.028	1.829
HCV-Ab	Positive (ref)	–	–	–	–
	Negative	1.058	0.784	0.707	1.584
HBsAb	Positive (ref)	–	–	–	–
	Negative	1.061	0.758	0.729	1.542
HBsAg	Positive (ref)	–	–	–	–
	Negative	0.667	0.339	0.291	1.530
CMV IgG	Positive (ref)	–	–	–	–
	Negative	1.814	0.384	0.474	6.947
Toxoplasma IgG	Positive (ref)	–	–	–	–
	Negative	1.158	0.486	0.766	1.749
CD4^+^ T cells (concurrently with SARS-CoV-2 infection)	< 33 percentile (ref)	–	–	–	–
	33–66 percentile	0.789	0.310	0.500	1.246
	> 66 percentile	0.864	0.518	0.555	1.346
CD8^+^ T cells (concurrently with SARS-CoV-2 infection)	< 33 percentile (ref)	–	–	–	–
	33–66 percentile	0.843	0.462	0.534	1.329
	> 66 percentile	1.081	0.732	0.691	1.692
CD4^+^/CD8^+^ (concurrently with SARS-CoV-2 infection)	< 33 percentile (ref)	–	–	–	–
	33–66 percentile	1.344	0.199	0.856	2.109
	> 66 percentile	0.829	0.419	0.527	1.305

*Ref* reference, *ART* antiretroviral therapy, *NRTI* nucleoside reverse transcriptase inhibitors, *INI* integrase inhibitors, *NNRTI* non- nucleoside reverse transcriptase inhibitors, *PI* protease inhibitors

*p* value in bold underlines the statistically significant association between variables

**Table 5:** Regression analysis to assess potential risk factors for PCCs onset in PLWH

Variable		Adjusted Odds ratio	p value	95%	CI
Sex	Female *(ref)*	–	–	–	–
	Male	**0.624**	**0.048**	**0.392**	**0.995**
Age, category	18–45 (ref)	–	–	–	–
	46–54	1.224	0.521	0.660	2.269
	55–59	1.763	0.083	0.929	3.349
	> 60	1.051	0.879	0.555	1.992
Ethnicity	Italians *(ref)*	–	–	–	–
	Foreigners	0.499	0.091	0.222	1.118
Years of HIV infection, category	*0–9 (ref)*	–	–	–	–
	10–19	0.687	0.206	0.384	1.229
	20–29	0.705	0.298	0.365	1.361
	> 30	0.674	0.269	0.335	1.356
Comorbidities	No comorbidities *(ref)*	–	–	–	–
	Comorbidities 1–2	1.264	0.323	0.794	2.013
	Comorbidities > 2	0.827	0.591	0.414	1.654
SARS-CoV-2 Vaccination	Unvaccinated *(ref)*	–	–	–	–
	Incomplete vaccination	0.672	0.274	0.331	1.368
	Complete vaccination	0.891	0.756	0.433	1.835
SARS-CoV-2 Infection Period	First period *(ref)*	–	–	–	–
	Second period	1.055	0.877	0.536	2.076
Hospitalization for COVID-19	No hospitalization *(ref)*	**–**	**–**	**–**	**–**
	Hospitalization	**1.904**	**0.054**	**0.988**	**3.668**
Nadir CD4^+^ T cells	< 33 percentile *(ref)*	–	–	–	–
	33–66 percentile	1.075	0.776	0.653	1.772
	> 66 percentile	0.952	0.868	0.536	1.694
HIV-RNA (concurrently with SARS-CoV-2 infection)	HIV-RNA < 20 cp/mL *(ref)*	–	–	–	–
	HIV-RNA 20–200 cp/mL	1.017	0.969	0.428	2.418
	HIV-RNA > 200 cp/ml	0.236	0.197	0.026	2.115
CD4^+^ T cells (concurrently with SARS-CoV-2 infection)	< 33 percentile *(ref)*	–	–	–	–
	33–66 percentile	0.717	0.240	0.411	1.249
	> 66 percentile	0.754	0.407	0.387	1.470
CD8^+^ T cells (concurrently with SARS-CoV-2 infection)	< 33 percentile *(ref)*	–	–	–	–
	33–66 percentile	0.875	0.638	0.502	1.527
	> 66 percentile	1.263	0.508	0.633	2.517
CD4^+^/CD8^+^ (concurrently with SARS-CoV-2 infection)	< 33 percentile *(ref)*	–	–	–	––
	33–66 percentile	1.699	0.073	0.952	3.029
	> 66 percentile	1.091	0.823	0.509	2.336

*Ref* reference

*p* value in bold underlines the statistically significant association between variables

## Discussion

In this retrospective, monocenter study, 17.1% (653/3817) of PLWH tested positive for SARS-CoV-2 via nasopharyngeal swab during the study period, and 34.9% (178/510) developed PCCs. Data obtained from the survey administrated by telephone interviews showed that the most commonly declared symptom 3 months after SARS-CoV-2 infection was fatigue (60/178; 33.7%). According to the regression analysis model, no variables influenced the PCCs risk in our cohort, including vaccination status or the period dominated by a specific SARS-CoV-2 variant. However, male subjects were found to be less likely to develop PCCs (adjusted OR: 0.62 IC 95% 0.39–0.99). Conversely, patients who were hospitalized for COVID-19 at the onset of infection appeared to be more likely to develop the PCCs (adjusted OR 1.90, 95% CI 0.98–3.66; p = 0.054). No patients reported PCCs after a second SARS-CoV-2 infection. Immunological (CD4^+^ cells, CD8^+^ cells, and CD4^+^/CD8^+^), virological (plasmatic HIV viremia), serological parameters for several infections (presence of CMV IgG, HCV antibodies, HBsAg, HBcAb or Toxoplasma IgG), and the number of comorbidities did not appear to alter the risk of PCCs in our cohort.

This study comprises a large number of PLWH with SARS-CoV-2 infection, covering the pre- and post-Omicron period and encompassing both vaccinated and unvaccinated patients against SARS-CoV-2. When the study was conducted, the Lombardy Region was the first and most heavily affected regions in Europe by the SARS-CoV-2 pandemic, beginning in February 2020 [11]. In January 2022, the Lombardy region faced a significant surge in SARS-CoV-2 cases attributed to the Omicron variant [12]. Despite a vaccination rate of approximately 90% (with at least one dose), this wave resulted in a higher number of infections, but fortunately a lower rate of deaths from the beginning of the pandemic [13]. Of course, the different virulence among the Omicron variant and the previously isolated might have had an influence.

Early in the pandemic, patients with frailty were identified as having a higher risk of short-term mortality from COVID-19 compared to non-frail patients. PLWH were also included in the context of frailty raising major concern regarding SARS-COV-2 impact on this population [14].

Brescia has one of the highest estimated incidence of HIV infection in Italy with a pre-pandemic rate of 5.8/100,000 compared to 4.7/100,000 in the whole country [15]. In our province, all PLWH are in follow-up in a single healthcare center: the Spedali Civili General Hospital of Brescia. During the early phases of the pandemic, an appropriate emergency response was mandatory to maintain the delivery of HIV care and protect our PLWH from COVID-19 [16]. As previously published, HIV infection was not associated with a higher risk of severe manifestations of SARS-CoV-2 during the acute phase compared to the general population in our Province [15]. Nonetheless, there is conflicting data in the literature regarding the severity of SARS-CoV-2 in PLWH, primarily because PLWH constitute a remarkably heterogeneous population in terms of immune competence, antiretroviral therapy coverage, and response to both and the SARS-CoV-2 vaccine [17, 18].

Most patients who recovered from acute COVID-19 experienced long-term effects on multiple organs and systems. Currently, there are no standardized criteria for diagnosing and categorizing post-COVID conditions, and the percentage of individuals reporting PCCs varies widely in the literature.

This variability can be attributed to several factors such as the time period, SARS-CoV-2 variant, patients included in the studies, geographical region, vaccination status, and the use of early therapies for COVID treatment [19–23]. Moreover, symptoms attributed to PCCs are often non-specific and may be due to other causes or concomitant morbidities. A nationwide Scottish population cohort study suggests that patients who had SARS-CoV-2 infection may mistakenly attribute their symptoms to long-COVID [24]. A debated topic is that SARS-CoV-2 infection could worsen a pre-existing inflammatory status, as chronic HIV infection [17]. Since in our cohort all the patients considered having PCCs fall into the WHO definition where post-COVID-19 conditions (symptoms of new onset, or if previously reported with a significant worsening, that could not be attributed to alternative causes [2]), we have not considered these symptoms as related to chronic HIV immune inflammation condition.

Much less data is available regarding PCCs in PLWH, with a variable prevalence ranging between 3%-78% [25]. Some studies investigated whether PLWH are at increased risk of PCCs compared to PLWH without SARS-COV-2 infection or with HIV-seronegative people with different results [8, 25–31]. In our cohort, the PCCs prevalence in PLWH was 35%, but the fact that HIV/AIDS might contribute to the PCCs symptomatology could not be ruled out, since we have not compared it to the PCCs prevalence among the general population in our setting. However, as highlighted in Table 3, an absence of statistical difference in viro-immunological characteristics of the two groups of PLWH (with and without PCCs) is reported. Given this, it is possible to infer that HIV/AIDS has not primarily affected the symptomatology of PCCs.

Comparing PLWH with and without SARS-CoV-2 infection, an elevated risk of multi-system dysfunction (i.e., respiratory, cardiovascular, and metabolic) was described among PLWH at 12 months post COVID-19 compared to PLWH without SARS-COV-2 infection [32]. Contrasting findings described how exercise capacity measured by a cardiopulmonary exercise testing was reduced among PLWH, but no differences are reported between SARS-CoV-2 infected and uninfected or among PLWH with or without PCC at 16 months after SARS-CoV-2 infection [33].

Yendewa et al. reported that PLWH had higher odds of PASCs, defined as persistence or occurrence of a new-onset health condition at least 28 days following COVID-19, compared to HIV-seronegative people and SARS-CoV-2 vaccination was protective [34]. Similar results were reported by Antar et al. who assessed the presence and severity of 49 long COVID-associated symptoms at 2 months following SARS-CoV-2 infection, although these findings were not consistent at 4–6 months post-infection [35].

The effectiveness of COVID-19 vaccines in preventing post-COVID conditions among vaccinated individuals remains uncertain, and it might vary depending on the number of vaccine doses received. A recent meta-analysis suggested that receiving a complete COVID-19 vaccination before contracting SARS-CoV-2 infection significantly reduces the risk of PCCs, even during the Omicron era. However, it did not protect against PCCs for those who received COVID-19 vaccination after COVID-19 infection (23). Since at the time of our study 46% of PLWH resulted unvaccinated, and 25% not completely vaccinated for COVID 19, the COVID vaccine may have played only a small role in reducing PCCs in our cohort. Furthermore, no statistical difference was seen between the PLWH with PCCs and without PCCs according to vaccine status. Therefore, we could infer that vaccination only had a marginal influence in our study.

Taken together, our results suggest that key variables related to HIV infection may not necessary increase the risk of PCCs, but they confirm some of the risk factors described in general population, such as gender, and severity of acute infection measured by hospitalization. One possible explanation could be that in our cohort of PLWH who completed the questionnaire, the majority had well-controlled HIV infection with plasma HIV RNA < 20 copies/ml (92%; 473/510), and a mean of CD4^+^ of 753 cells/mm3. Additionally, 65% (334/510) did not have any comorbidities, and 53% (274/510) had received at least 2 doses of COVID-19 vaccine at the time of SARS-CoV-2 infection. Moreover, 60% of them (309/510) acquired SARS-COV-2 infection during Omicron wave. Further studies are needed in different cohorts and settings to confirm our findings.

The main limitations of our study include the absence of a control group comprising individuals without HIV infection, or PLWH without SARS-CoV-2 infection, the collection of symptoms was performed via telephone interview and the lack of evaluation regarding the use of COVID therapies, including antiviral drugs. Additionally, people with mild symptoms may have been less likely to undergo SARS-CoV-2 testing. Moreover, Omicron wave surge coincides with the inoculation of the 3rd dose of SARS-CoV-2 vaccine during the Italian vaccination campaign, hence eventual adverse effects of the vaccine considered as PCCS in fully vaccinated PLWH could not be ruled out. However, we did not observe any statistical difference among PLWH with PCCs and without PCCs according to the vaccination status (unvaccinated, incomplete or complete vaccination). Strengths of this study include the large sample size and the linkage with the local health service, which provided all notifications of SARS-CoV-2 test results performed in the Brescia Local Health Agency.

## Supplementary Information

Below is the link to the electronic supplementary material.

Supplementary file1 (DOCX 22 KB)

## Data Availability

Not applicable.
