# Supplementary material for: Long-term symptoms after SARS-CoV-2 infection in a cohort of people living with HIV
Source: Infection. 2024 May 3;52(6):2339–50. doi: 10.1007/s15010-024-02288-9 (PMC11621153; doi:10.1007/s15010-024-02288-9)
Supplement: Supplementary file 1 — Supplementary file1 (DOCX 22 KB) [file 15010_2024_2288_MOESM1_ESM.docx]

**Supplementary Material:** Telephone questionnaire administered to PLWH with symptoms associated with long-COVID. The symptoms were recorded for each patient considering the onset (within 4 weeks, between 4 and 12 weeks and beyond 12 weeks post SARS-CoV-2 infection).

| LONG-COVID SYMPTOMS | Onset <4 weeks after acute SARS-CoV-2 infection | Onset between 4-12 weeks after acute SARS-CoV-2 infection | Onset >12 weeks after acute SARS-CoV-2 infection |
| --- | --- | --- | --- |
| GASTROINTESTINAL | | | |
| - Dysgeusia/ageusia |  |  |  |
| - Parosmia/anosmia |  |  |  |
| - Loss of appetite |  |  |  |
| - Dysphagia |  |  |  |
| - Nausea/vomit |  |  |  |
| - Weight loss/gain |  |  |  |
| - Diarrhoea |  |  |  |
| - Constipation |  |  |  |
| RESPIRATORY | | | |
| - Exertional dyspnea |  |  |  |
| - Dyspnea at rest |  |  |  |
| - Palpitations |  |  |  |
| - Voice alteration |  |  |  |
| - Sore throat |  |  |  |
| - Cough |  |  |  |
| - Rhinitis |  |  |  |
| NEUROPSYCHOLOGICAL | | | |
| - Insomnia |  |  |  |
| - Mental confusion |  |  |  |
| - Mood disorders |  |  |  |
| - Agitation |  |  |  |
| - Anxiety attacks |  |  |  |
| - Concentration problems |  |  |  |
| - Memory alterations |  |  |  |
| - Headache |  |  |  |
| - Neuralgia |  |  |  |
| - Speech disorders |  |  |  |
| - Transient changes in consciousness/seizures |  |  |  |
| - Episodes of lipothymia/syncope |  |  |  |
| - Trembling |  |  |  |
| - Dizziness |  |  |  |
| - Asthenia |  |  |  |
| OSTEOMUSCOLAR | | | |
| - Decrease in strenght |  |  |  |
| - Muscolar pain |  |  |  |
| - Changes in muscle sensitivity |  |  |  |
| OTHERS | | | |
| - Fever |  |  |  |
| - Ocular disorders |  |  |  |
| - Lower limb oedema |  |  |  |
| - Hair loss |  |  |  |
| - Decreased libido |  |  |  |
| - Dermatological disorders |  |  |  |
